# Sustainable Drug Delivery of Famotidine Using Chitosan‐Functionalized Graphene Oxide as Nanocarrier

**DOI:** 10.1002/gch2.201900002

**Published:** 2019-08-14

**Authors:** Chetan Ramesh Mahajan, Lalit B. Joshi, Umakant Varma, Jitendra B. Naik, Vijay Raman Chaudhari, Satyendra Mishra

**Affiliations:** ^1^ University Institute of Chemical Technology North Maharashtra University Jalgaon 425001 Maharashtra India

**Keywords:** chitosan, famotidine, graphene oxide, in vitro drug release, sustainable drug delivery

## Abstract

This work mainly focuses on the graphene oxide (GO)‐assisted sustainable drug delivery of famotidine (FMT) drug. Famotidine is loaded onto GO and encapsulated by chitosan (CH). UV‐visible spectroscopy, field emission scan electron microscopy, and atomic force microscopy confirm the loading of FMT on GO. An interaction of FMT with GO and CH through amine functionalities is confirmed by Fourier‐transform infrared spectroscopy. Differential scanning calorimetric and cyclic voltammetric investigations confirm the compatibility of FMT and its retaining activity within chitosan‐functionalized graphene oxide (CHGO) composite. Encapsulation efficiency of FMT is determined for various CHGO‐FMT combinations and found to be higher at 1:9 ratio. The in vitro drug release profile is studied using a dissolution test apparatus in 0.1 m phosphate buffer medium (pH = 4.5), which shows sustainable drug release up to 12 h, which is greater than the market product (Complete release within 2 h). Comparative study of drug encapsulated with CH and without GO elucidates that GO is responsible for the sustainable release. The “*n*” value obtained from slope using Korsmeyer–Peppas model suggests the super case‐II transport mechanism.

## 1 Introduction

Natural biopolymers showed the potentiality in control drug delivery due to their nontoxicity, biocompatibility, renewability, biodegradability, and environmental sensitivity.^1^ Some of the examples of biopolymers are starch, gum,^2^, ^3^, ^4^, ^5^, ^6^ chitosan (CH),^7^, ^8^, ^9^, ^10^ konjac glucomannan,^11^, ^12^, ^13^ and sodium alginate.^14^, ^15^ Unfortunately, they suffered with the lacunae such as weak mechanical properties and burst releases of drugs and hence failed to avoid completely the intensive adverse side effects of drug therapy. Therefore, it is an utmost need to improve further drug therapeutic action. Some researchers worked on improvement of mechanical properties of chitosan with the help of inorganic nanofillers.^16^, ^17^ Many researchers explored newer carrier systems such as metal and metal oxide nanoparticles,^18^, ^19^, ^20^ polymeric micelles,^21^, ^22^, ^23^ liposome's,^24^, ^25^, ^26^, ^27^ dendrimers,^28^, ^29^ and carbon nanotube,^30^, ^31^ as targeting or controlling agents for expected drug release profile. Required characteristics of the carrier are binding sites for drug, biocompatibility, nontoxic nature, site specificity, safer elimination, and improved drug solubility.^32^ Recent discovery of graphene oxide (GO) attracted the researcher's attention due to its potentiality to provide most of the aforementioned characteristics of carrier for drug delivery.^33^ Graphene, a single layer of sp^2^‐hybridized carbon atoms arranged in a honeycomb 2D crystal lattice, possesses remarkable physicochemical properties, including a high Young's modulus, high fracture strength, large specific surface area with provision to tune the surface chemistry, nontoxicity, and biocompatibility.^34^, ^35^, ^36^ Hence, graphene can play significant role to overcome the challenges in recent drug therapy. GO is a graphene sheet decorated with oxygen‐containing functional groups such as hydroxyl and epoxy groups on the basal planes and carbonyl and carboxylic groups at the edges. Moreover, atomically thick sheet like structure provides the maximum specific surface area to load and carry the drug via surface interaction. These properties project the GO as an ultimate material for variety of biomedical applications. Researchers proved GO as a drug binding candidate for hydrogel functionalization in drug delivery as well as biocompatibility to living cells that render its state of the art use drug delivery systems.^20^ Degradation of GO has not been reported yet due to hard backbone network of GO while it is well reported as biocompatible after its cytotoxicity studies.^37^

Famotidine (FMT) is a histamine H_2_ receptor antagonist agent used for treatment of ulcer in stomach, gastroesophagus reflux, esophagitis, etc., which has oral bioavailability of 40–45%.^38^ Recent route of FMT administration consequences the side effects such as anaphylaxis cause long QT syndrome, complete atrioventricular block and cardiac arrest, etching,^39^, ^40^, ^41^ etc. In order to avoid these side effects, it is required to pay attention to control the release profile of FMT for desired therapeutic action. The biodegradable polymers are widely used to get sustained release profile of drug. Drug encapsulation with polymer is very important parameter, but sometimes drugs are unable to bind with the polymer due to less amount of binding fictional sites. Therefore, to avoid this problem, there is need to develop or create the drug carrier with more efficient nature.

Many researchers tried to invent newer carrier system for drug delivery using magnetic nanoparticle,^42^, ^43^ hydrophilic colloids,^44^, ^45^ hydrogel,^46^ microsphere,^47^, ^48^, ^49^ lipid solid dispersion,^50^, ^51^ effervescent floating tablet,^52^ alginate beads,^53^, ^54^ silica nanoparticles,^55^, ^56^ pronisomes,^57^ etc. However, compared with them, GO shows most of the features required for ideal drug carrier and hence can be a potential alternative to the reported carriers for the control drug release.

Wei and co‐workers^58^ worked on biofunctionalization of GO for drug delivery application and explained the interaction of biomolecules with graphene. They developed and investigated the covalent‐interaction‐mediated assembly of thermosensitive polymer nanoparticles (PNPs) on functionalized GO nanosheets to create novel GO–PNP hybrids for drug delivery. GO nanosheets provide reactive sites for the binding of PNPs. GO–PNP hybrids were prepared by the covalent interaction mediated assembly and characterized. The drug molecules were loaded with GO–PNP which showed greater improvement than PNPs.^59^ The chemical modification in graphene material for designing novel functional monohybrids will played key role in drug delivery and also applicable in other biomedical fields, such as biosensing and tissue engineering.^60^

In present work, we aimed to design the FMT formulation in conjunction with CH functionalized GO, to achieve the desired sustainability in drug delivery. GO functionalized with CH for provide the maximum functional binding sites to FMT. As per our knowledge, this is first report for sustain drug release profile for FMT using GO as nanocarrier. The significance of this research work is that FMT drug formulation shows sustainable release behavior with longer therapeutic action for treatment and overcome the repeated dosing.

## 2 Characterization

Chemical nature of prepared composite materials was investigated using Fourier transform infrared (FTIR) spectroscopy (Shimadzu‐8400 spectrometer, Japan) within the frequency range of 4000 to 400 cm^−1^. Reported spectra were averaged of 100 scan with the resolution of 2 cm^−1^.

Raman spectra were recorded using Olympus BX41 microscope having JOBIN YVON HORIBA HR 800 UV detector with 2 mW power and organ laser wavelength 514.5 nm using lens X10.

Amount of FMT encapsulated in composite was determined by UV‐visible spectrophotometer (Cary60 UV–vis spectrophotometer). The encapsulation efficiency (EE) of FMT was calculated using following equation

(1) $$\%\text{EE}\;\;=\;\;\frac{\text{Weight}\;\;\text{of}\;\;\text{drug}\;\;\text{in}\;\;\text{complex}}{\text{Theoretical}\;\;\text{weight}\;\;\text{of}\;\;\text{drug}}\;\;\times \;\;100$$

Crystalline nature was judged by X‐ray diffractometer (XRD), Bruker, D8 ADVANCE (Bruker Corporation, Tokyo, Japan) using monochromatic Cu *K*_α_ radiation (λ = 1.5406 Å) at 40 kV and 40 mA. Scan rate was 5° min^−1^ between the angles 5° and 80°.

Surface morphology was observed using field emission scanning electron microscope (FE‐SEM), HITACHI S‐4800, operated at 5–15 Kv. Suspension was drop casted onto carbon tape and dried at room temperature. Prior to analysis, sample was coated with gold to avoid degradation or burning due to high power. Energy dispersive X‐ray spectroscopy (EDS) attached with FE‐SEM was used for elemental analysis.

Topographical morphology was observed using atomic force microscope (AFM) TriA100 scanning probe microscope system, APE Research Nanotechnology, Italy, containing noncontact mode. The tip was used of the mMasch (HQNSC15) having length 125 mm, width 30 mm, and thickness 4 mm with resonance frequency 325 KHz and Force constant was 40 N m^−1^.

Differential scanning calorimeter (DSC, Perkin Elmer DSC 4000, Netherland) thermograms were recorded by loading sample into crimped aluminum pans and heated between the temperature ranges from 20 to 600 °C at a heating rate of 10 °C min^−1^ under dry N_2_ gas (25 mL min^−1^). Melting temperature was taken as the peak of the melting endotherm. The error in each measurement was estimated to be ±0.5 °C. Instrument was calibrated using metallic indium (99.9% purity).

Standard stock solution containing FMT was prepared by dissolving 10 mg of FMT in phosphate buffer pH = 4.5 in 100 mL volumetric flask. It was then sonicated for 10 min and then final volume was made up to 100 mL to get stock solution containing 100 µg mL^−1^. Solutions scanned in the range of 200–400 nm region by using U‐2900 Hitachi spectrophotometer. Accurate volumes were transferred into set of 10 mL calibrated volumetric flasks. The series contain varying concentration of FMT (0–10 µg mL^−1^) was prepared. The calibration curve was constructed by plotting drug concentration versus absorbance values at 265 nm for FMT, and regression equation was determined.

### 2.1 % EE by UV

The amount of FMT encapsulated into the CHGO‐FMT was determined by UV–vis spectrophotometer. An accurately weighed 10 mg FMT of CHGO‐FMT was stirred with methanol (5 mL) and 2% acidic acid (5 mL) to dissolve the polymeric coat and extracted in phosphate buffer solution (pH = 4.5). Stirring was continued for 30 min to facilitate the evaporation of organic solvent. The dispersion was filtered and the residue was washed with phosphate buffer solution. The % EE was determined in the filtrate after appropriate dilution with phosphate buffer solution by UV at 265 nm.

Cyclic voltammograms were recorded using three electrode system consisted of platinum wire, Ag/AgCl (3 m KCl), and functionalized glassy carbon (GC) as counter, reference and working electrodes, respectively. 0.1 m phosphate buffer solution (pH = 4.5) was used as electrolytic medium. Known amount of respective dispersion was casted on GC. Nafion was used as binder to avoid the film fouling.

### 2.2 In Vitro Drug Release

Drug release profile was evaluated using Dissolution Test Apparatus, Type‐II (Paddle method, Electro lab, TDT 06) at 37 ± 0.5 °C and at paddle speed of 100 rpm. The release studies were performed in 900 mL capacity bowl having the phosphate buffer medium (0.1 m, pH = 4.5). 5 mL aliquot was withdrawn from the dissolution apparatus at regular time intervals of 1 h and filtered through a membrane filter (0.45 µm). The withdrawn sample was replenished with 5 mL of fresh media to maintain the sink condition. Drug content was determined with the help of double beam UV‐visible spectrophotometer (U‐2900, Hitachi, Japan) by measuring absorbance at 266 nm wavelengths. To study the mechanism of drug release from the carrier system, zero order, first order, Higuchi equation and Korsmeyer–Peppas equation were selected as a model dependent approach to characterize the dissolution profile.^61^, ^62^, ^63^ These selected models are often used to describe the drug release from the polymeric system when the mechanism is not well known or when more than one type of release phenomenon is involved. The model which gave the highest coefficient of determination (*R* ^2^) was considered to be the most suitable kinetic model for describing the release of FMT from the carrier system.

## 3 Results and Discussion

**Figure 1:**
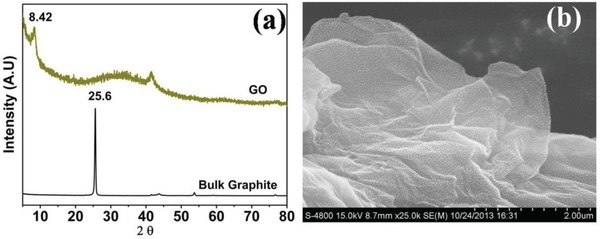
a) X‐ray diffractogram and b) FE‐SEM image of GO.

In XRD (**Figure** **1**a), (001) and (100) diffraction peaks observed at 8.4° and 43.3°, respectively, which confirm the exfoliation of graphitic sheet upon oxidative treatment.^64^, ^65^, ^66^ The (001) and (100) planes showed that graphene oxide has hexagonal structure with sp^2^ bonded carbon.^67^ Interlayer sheet spacing between graphene sheet in GO was observed to be 1.04 nm, determined from the (001) crystalline plane. FE‐SEM image in Figure 1b clearly shows the sheet like structure with approximate dimensions of few µm^2^. Elemental analysis performed using EDS estimated the concentration of carbon and oxygen as 70.9% and 28.3%, respectively.

**Figure 2:**
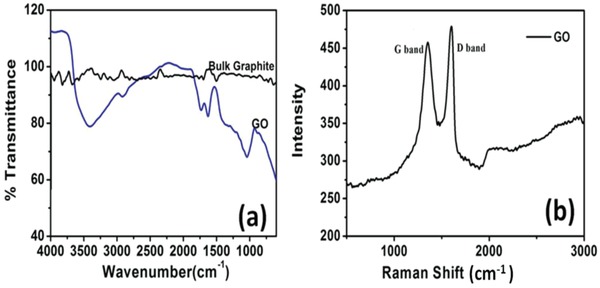
a) FTIR and b) Raman spectra of GO.

Vibration bands observed at 3412, 1731, and 1042 cm^−1^ in FTIR spectrum of GO (**Figure** **2**a) correspond to the O—H, C=O, and C—O bonds, respectively.^8^, ^68^ Raman spectrum shows the presence of D‐band and G‐band at 1352 and 1602 cm^−1^, respectively (Figure 2b). Presence of D‐band indicates the defective structure of GO due to the presence of oxygenated functional groups. Despite of functionalization, intensity ratio intensity of I band/intensity of G band (1.05) was more than one and indicated the good quality of GO as compared to reported ones.^64^, ^65^, ^68^, ^69^

**Figure 3:**
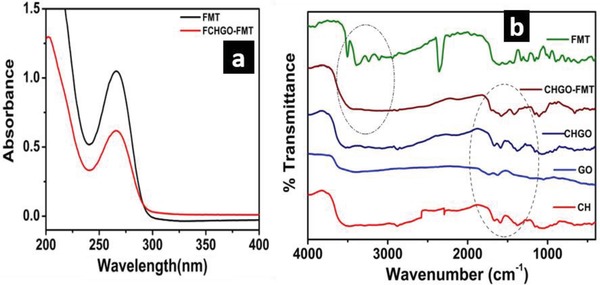
a) UV‐visible spectra of aqueous solution of FMT and FMT loaded in GO‐CH nanocomposites. b) FTIR spectra of CH, GO, CHGO, CHGO‐FMT, CH‐FMT, and FMT.

The synthesized GO was used as carrier for FMT drug delivery in association with CH as an encapsulating agent. To elucidate the role of GO toward FMT drug delivery, control sample was prepared with encapsulation of FMT without GO. Presence of FMT in prepared nanocomposite was observed by recording the UV–vis spectra as depicted in **Figure** **3**a. Pure FMT showed the peak in UV‐visible spectrum ≈266 nm, which was also observed at same position in spectrum recorded for FMT loaded nanocomposite, which confirms the loading of drug.

**Figure 4:**
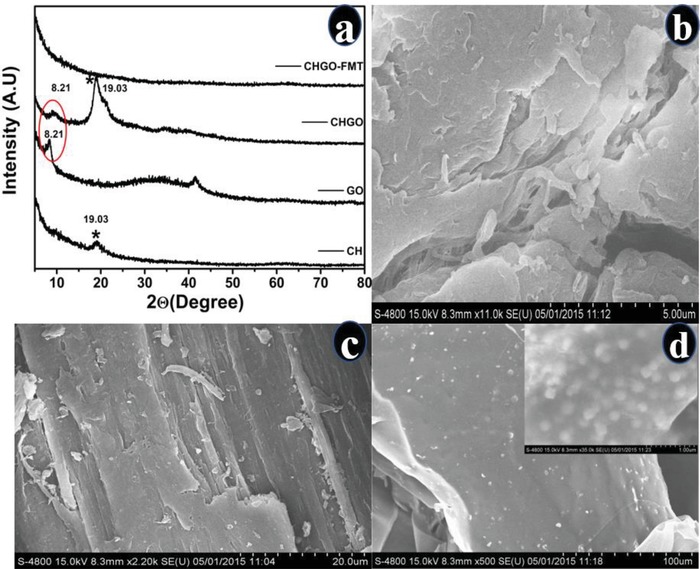
a) X‐ray diffractogram of CH, GO, CHGO composite, and CHGO‐FMT. FE‐SEM image of b) CH, c) CHGO, and d) CHGO‐FMT.

Nature of interaction within GO of CH and FMT was observed by FTIR spectroscopy (Figure 3b). After encapsulation of GO with CH, carbonyl stretching frequency in GO (1744 cm^−1^) was disappeared completely with emergence of new bands at 1682 and 1591 cm^−1^, which suggested the amide linkage between GO and CH.^31^, ^70^ FTIR spectrum of FMT showed vibration bands for amines (3506, 3400, and 3240 cm^−1^), imines (2343 cm^−1^) and S=O (1147 cm^−1^). However, after encapsulation by CHGO, bands arised due to amines and imines were disappeared. This indicated that FMT interacts with CHGO through amine and imine functionality. Former interaction can be understood due to the presence of C=O bonds in CHGO; however, later one can be supported by sp^2^ character of GO present into composite. Such interaction was absent for CH‐FMT composite (without GO), suggested that GO had provided additional interacting sites for drug. Hence, FTIR spectra clearly demonstrated the encapsulation of FMT within CHGO composite through amine and imine interaction. FMT encapsulation was further evaluated through XRD and FE‐SEM as shown in **Figure** **4**a–d.

XRD diffraction patterns were observed at 8.4° and 19° for GO and CH, respectively (Figure 4a), correspond to the (001) plane of GO and CH, respectively.^58^, ^71^ Addition of GO caused increase in the crystallinity of CH due to reinforcement effect that provided sharply intense peak at 19°. In case of CHGO, both the peaks of CH as well as GO are present; however, they disappeared after loading of FMT. Absence of CH peak can be justified on the basis of amorphous nature of CH due to the lyophillization of the product. However, diminishing of GO peak can be attributed to the insertion of FMT within GO layers which leads to expansion of sheet.

On comparing the FE‐SEM images of CH, CHGO, and CHGO‐FMT (Figure 4a–d), It is clearly indicated that the CH has uniform surface (4b), while in case of CHGO the CH has adhered on to GO sheets (4c). The formation of expanded and wrapped sheet like structure after loading of FMT was observed (4d). Hence, loading of FMT resulted in an expansion of GO sheets, which was also noticed in XRD. Therefore, based on FTIR, XRD, and FE‐SEM, it can be concluded that FMT was encapsulated into CHGO composite. Besides, FMT interacts with composite through amine and imine bonds along with expansion of GO sheets.

**Figure 5:**
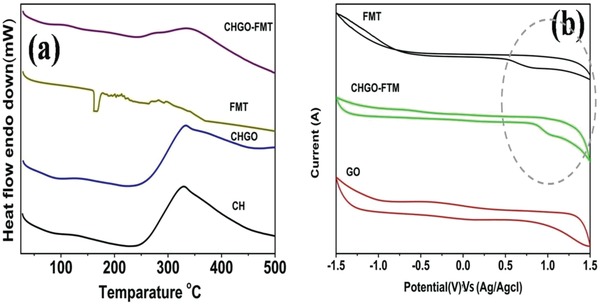
a) Differential scanning calorimetric graph CH, CHGO composite, FMT, and CHGO‐FMT. b) Cyclic voltammograms recorded in 0.1 m phosphate buffer solution (pH = 4.5) using GO, CHGO‐FMT, and FMT deposited on glassy carbon electrode.

Compatibility of FMT within composite was observed through differential scanning calorimetery (DSC) and cyclic voltammetry as shown in **Figure** **5**.

Melting temperature of CH was observed to be 350 °C for isolated state, in composite form with GO and after FMT loading too. Moreover, peak corresponds to FMT was also absent for the CHGO‐FMT (Figure 5a). These observations indicated that CH and FMT were compatible within the composites.

We further investigated the behavior of FMT within composite through cyclic voltammetric investigations (Figure 5b). Similar nature of oxidation behavior of FMT was observed for pure FMT and FMT within composite, which indicated the presence and retained behavior of FMT within composite. However, 0.2 V shifting of FMT oxidation potential for composites can be attributed to the physical barrier created by GO within composite.

**Figure 6:**
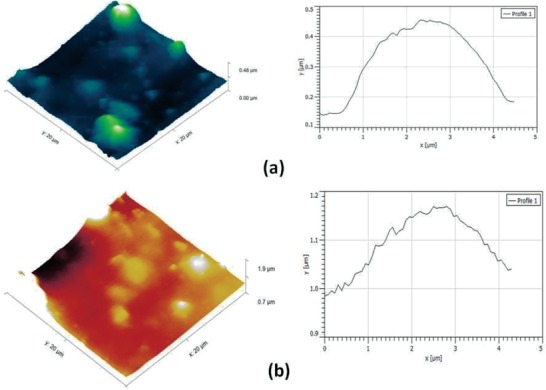
AFM image of a) CHGO and b) CHGO‐FMT.

According to the AFM images, CHGO contains CH functionalized GO which had a thickness of 0.48 µm (**Figure** **6**a); it was less than modified CHGO with FMT, i.e., 1.9 µm (Figure 6b). The improvement in the thickness of CHGO‐FMT was happened due to CHGO arranged in layer by layer fashion and encapsulated the FMT in layers. In Figure 4d, FE‐SEM of CHGO‐FMT showed the clear evidence that layer by layer arrangement of CHGO is capable of adsorbing FMT compounds. The FMT has been adsorbed via inter/atomic interactions in between layers of CHGO. The functionalized nature of CHGO is very useful for drug carriers. There are few reports published on the controlled/sustained released targeted delivery with different drugs of two or more different drugs using graphene‐based nanocarrier.^70^, ^72^, ^73^

EE of drug at given dose and varying polymer ratio was evaluated from absorbance of drug using Equation (1) and included as **Table** **1**. As the drug to carrier ratio was increased, EE also increased simultaneously, except for last ratio of 1:11. This is because of achieving the saturation point of drug encapsulation by CH matrix. Highest EE was observed at ratio of 1:9. Comparative study depicts that in case of CHGO‐FMT, this increment was quite high as compared to CH‐FMT; and GO is responsible for the 15% increase in EE than CH.

**Table 1:** Encapsulation efficiency (EE) of famotidine within nanocomposites prepared at various proportions

Drug to carrier ratio	CH‐FMT [%]	CHGO‐FMT [%]
1:1	41.8	48.8
1:3	46.5	52.2
1:5	45.6	58.3
1:7	49.5	60.1
1:9	52.2	67.2
1:11	40.7	49.5

CH has a high biocompatibility and biodegradability, nontoxic in nature, low immunogenicity, inherent antibacterial properties, very good solubility due to hydrophilic nature, and widely use as pharmaceutical active drug carrier material for sustainable drug delivery property. CH has prominent functional groups which get efficiently functionalized GO. CHGO provides large amount of functional binding spots to FMT and gives biocompatibility. GO was functionalized with CH that gives the sustainable delivery of FMT as well as CH helps to encapsulate FMT and that will help to release it at targeted region. These properties result in promising platform for drug carrier mechanism and advanced drug delivery systems.

### 3.1 In Vitro Drug Release

**Figure 7:**
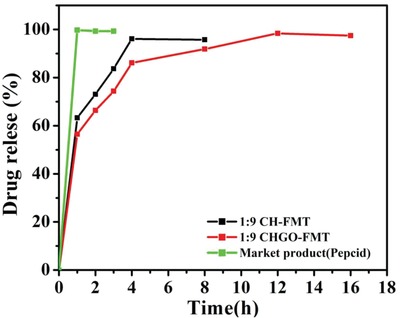
Drug release profile of famotidine obtained for pure famotidine procured from market. Famotidine loaded on graphene oxide and encapsulated with CH in 1:9 famotidine: composite ratio (CHGO‐FMT) and CH encapsulated famotidine (CH‐FMT).

In vitro drug release study was carried out for the CHGO‐FMT having higher EE (**Figure** **7**), which showed an excellent sustained release for FMT loaded on CHGO‐FMT composite. Almost half quantity (i.e., 56%) of drug released within first hour which is advantageous for quick therapeutic activity. Highest amount of drug released (98%) is observed for 12 h span which is quite sustainable as compared to the FMT loaded into CH (without GO) and FMT obtained from market. Relatively faster release profile of drug during an initial hour may be from surface anchored drug particulates those get dissolved immediately as compared to the core level encapsulated drug which takes time to dissolve. Results clearly indicate that GO is responsible for the improved sustainability of the FMT.

**Figure 8:**
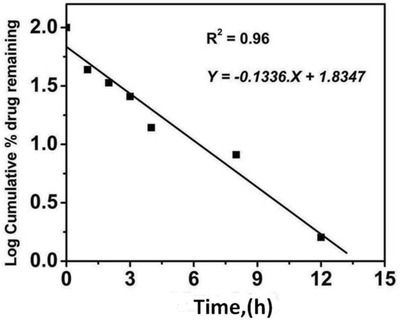
Fitting of drug release profile data into first order Kinetic model.

Release mechanism was investigated by fitting the data with different kinetic models (Zero order, First order, Matrix, Peppas, and Hixson–Crowell).^62^ The data reliably fitted into first order equation (**Figure** **8**) with appreciable linearity (*R* ^2^: 0.96). Release mechanism was studied using Korsmeyere–Peppas model. First 60% of drug release was fitted in Korsmeyere–Peppas model to determine the release exponent “*n*” which is indicative of drug release mechanism. According to Korsmeyer theory, if “*n*” is 0.45 then drug release will follows Fickian diffusion mechanism; for 0.45 < *n* < 0.89 follows Anomalous (non‐Fickian) diffusion; for *n* = 0.89 case II transport and for *n* > 0.89 diffusion mechanism will super case II transport.^74^

The “*n*” obtained by Korsmeyer–Peppas model is found to be more than 0.89 which suggests the super case‐II transport, i.e., drug releases by both diffusion and relaxation of the GO sheets. FMT exhibits super case‐II transport as dominated mechanism for optimized formulation.^75^ Kinetics and mechanisms of FMT release from CH‐FMT were investigated as shown in Figure S1 in the supporting Information. The data reliably not fitted into first order equation with appreciable linearity.

## 4 Conclusion

FMT drug was loaded onto GO and encapsulated by CH. The drug showed compatibility and chemical interaction with GO based composite. Encapsulation efficiency of FMT was higher at 1:9 ratio of FMT: CHGO. In vitro release, profile depicted the sustainable drug release extended up to 12 h as compared to the market product (complete release within 2 h). FMT‐CHGO also showed 56% release within initial hour, an important aspect for quick therapeutic treatment. Comparative study confirmed the reasonable contribution of GO toward sustainable release of FMT. Release profile data followed the first order kinetic model and obtained slope value using Korsmeyer–Peppas model suggested the super case‐II transport.

## 5 Experimental Section

*Materials and Method*: Graphite fine powder (extra pure) was procured from Loba chemie Pvt Ltd, (Mumbai, India). Sulfuric acid 90%, orthophosphoric acid (85%), hydrogen peroxide, hydrazine hydrate, acetic acid (99.9%), and ether were procured from Merk specialty Pvt. Ltd. (Mumbai, India). CH extracted from shrimp shells (with a degree of deacylation of ≥75% estimated by titration methods) was used as procured from Sigma‐Aldrich, USA. *N*,*N*‐dimethylformamide (DMF, 99.8%), *N*,*N*′‐dicyclohexylcarbodiimide (DCC, 99.0%), 1,2‐dichlorobenzene (ODCB, 99.0%), and 4‐dimethylaminopyridine (DMAP, 99.0%) were purchased from Arva synthesis Pvt. Ltd. (Mumbai, India). All reagents were used without further purification.

*Synthesis of Graphene Oxide*: GO was prepared through improved Hummer's method.^68^, ^76^, ^77^ Typically, 9:1 mixture of concentrated H_2_SO_4_ and H_3_PO_4_ was added in reaction assembly containing graphite powder (3 g). KMnO_4_ (sixfold wt. equivalent to graphite powder) was added slowly to the above reaction mixture and heated at ≈50 °C for 12 h. Reaction mixture was cooled to room temperature and poured onto ice (400 mL) followed by addition of 30% H_2_O_2_. Suspension was filtered through polyester fiber cloth and the filtrate was centrifuged at 4000 rpm. Obtained solid product was washed twice subsequently with copious amount of double distilled water, 30% HCl and ethanol (200 mL). Resultant material was coagulated with 200 mL of petroleum ether. Obtained solid material was vacuum‐dried overnight at room temperature and stored under vacuum till further use.

*Preparation of Chitosan Functionalized Graphene Oxide (CHGO)*: 50 mg of GO and 1000 mg CH were mixed with 50 mL DMF in 100 mL capacity round‐bottom flask and sonicated for 1 h. 450 mg DCC and 300 mg DMAP were then added to the above suspension and incubated for 48 h at room temperature. Resulting solid was isolated by centrifugation and washed with ODCB (3 × 50 mL) to remove unreacted chitosan. The mixture was subsequently washed thoroughly with water, methanol, and acetone, and finally dried under vacuum at 60 °C for 24 h.^70^, ^77^

*Preparation of CHGO‐FMT Composite*: CHGO and FMT were solubilized separately in 2% acetic acid through sonication and stirring. Solution of FMT was added dropwise to the CHGO suspension with constant stirring to form a homogeneous mixture. After complete addition, mixture was lyophilized for 24 h and named as *CHGO‐FMT*. To investigate the role of GO, FMT was loaded on chitosan excluding GO using the procedure followed for preparation of CHGO‐FMT as comparative batches. Formulation of CHGO‐FMT was also tuned by varying the ratio of chitosan: GO to obtain the maximum loading of FMT.

## Conflict of Interest

The authors declare no conflict of interest.

## Supporting information

Supplementary
