# Supplementary material for: Sustainable Drug Delivery of Famotidine Using Chitosan‐Functionalized Graphene Oxide as Nanocarrier
Source: Glob Chall. 2019 Aug 14;3(10):1900002. doi: 10.1002/gch2.201900002 (PMC6777207; doi:10.1002/gch2.201900002)
Supplement: Supplementary file 1 — Supplementary [file GCH2-3-1900002-s001.pdf]

Copyright WILEY-VCH Verlag GmbH & Co. KGaA, 69469 Weinheim, Germany, 2019.

Global Challenges
Open Access

Supporting Information

for *Global Challenges,* DOI: 10.1002/gch2.201900002

Sustainable Drug Delivery of Famotidine Using Chitosan-Functionalized Graphene Oxide as Nanocarrier

*Chetan Ramesh Mahajan, Lalit B. Joshi, Umakant Varma, Jitendra B. Naik, Vijay Raman Chaudhari, and Satyendra Mishra**

Sustainable Drug Delivery of Famotidine using Chitosan- Functionalized Graphene Oxide as Nano-carrier

Chetan R. Mahajan, Lalit B. Joshi, Umakant Varma, Jitendra B. Naik, Vijay Raman Chaudhari, Satyendra Mishra*

University Institute of Chemical Technology, North Maharashtra University, Jalgaon 425001, Maharashtra, India.

[*] Corresponding author: profsm@rediffmail.com,

Ph: +91 257 225842, Fax: +91-257-225840

Table S1: Amount of FMT encapsulated within the CHGO samples (at encapsulation efficiency of 67.2 %)

Characterization	Amount of CHGO (mg)	Amount FMT encapsulated (mg)
XRD	10	0.75
FTIR	2	0.14
SEM	1	0.07
DSC	5	0.37

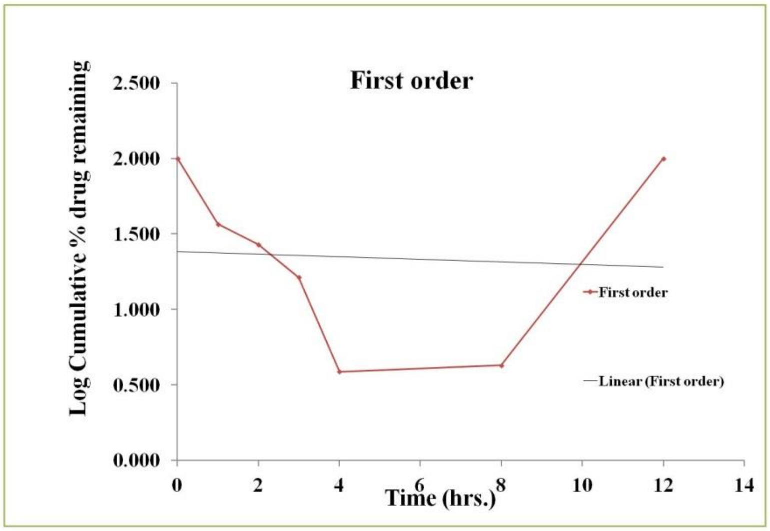

Figure S1 Kinetics and drug release mechanism of CH-FMT
